# Supplementary material for: Efficient metal halide perovskite light-emitting diodes with significantly improved light extraction on nanophotonic substrates
Source: Nat Commun. 2019 Feb 13;10:727. doi: 10.1038/s41467-019-08561-y (PMC6374404; doi:10.1038/s41467-019-08561-y)
Supplement: Supplementary file 1 — Supplementary Information [file 41467_2019_8561_MOESM1_ESM.pdf]

Efficient metal halide perovskite light-emitting diodes with significantly improved light extraction on nanophotonic substrates

Zhang *et al.*

Efficient metal halide perovskite light-emitting diodes with significantly improved light extraction on nanophotonic substrates

Qianpeng Zhang,[1,2] Mohammad Mahdi Tavakoli,[1,] Leilei Gu,[1,2] Daquan Zhang[1], Lei Tang,[1] Yuan Gao,[1] Ji Guo,[1] Yuanjing Lin,[1] Siu-Fung Leung,[1,†] Swapnadeep Poddar,[1] Yu Fu[1] and Zhiyong Fan[1,2,*]

[1]Department of Electronic and Computer Engineering, The Hong Kong University of Science and Technology, Clear Water Bay, Kowloon, Hong Kong SAR, China

[2]HKUST-Shenzhen Research Institute, No. 9 Yuexing first RD, South Area, Hi-tech Park, Nanshan, Shenzhen 518057, China

[†]*Present Address:* Department of Computer, Electrical and Mathematical Sciences and Engineering, King Abdullah University of Science and Technology, Thuwal 23955-6900, Kingdom of Saudi Arabia

[*]email: eezfan@ust.hk

Supplementary Figures

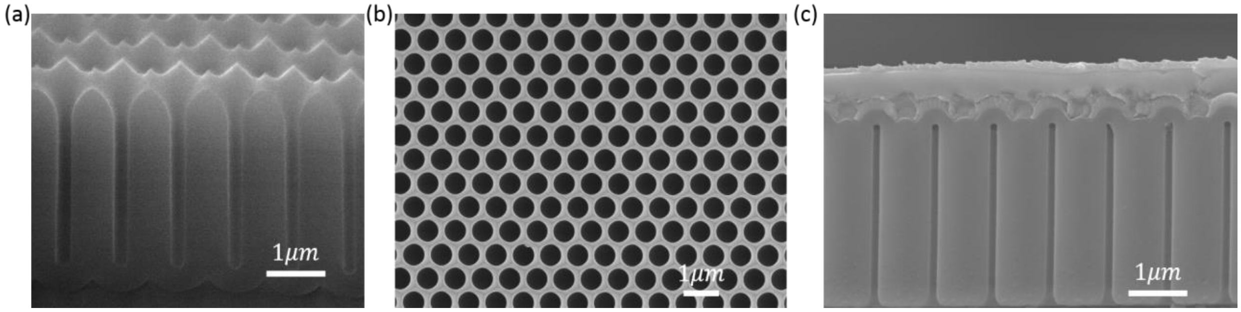

Supplementary Figure 1. (a) Cross-sectional and (b) top view SEMs of imprinted 400 V AAM with 1000 nm pitch. (c) Cross-sectional view of device fabricated on 1000 nm pitch AAM w/o TiO_2.

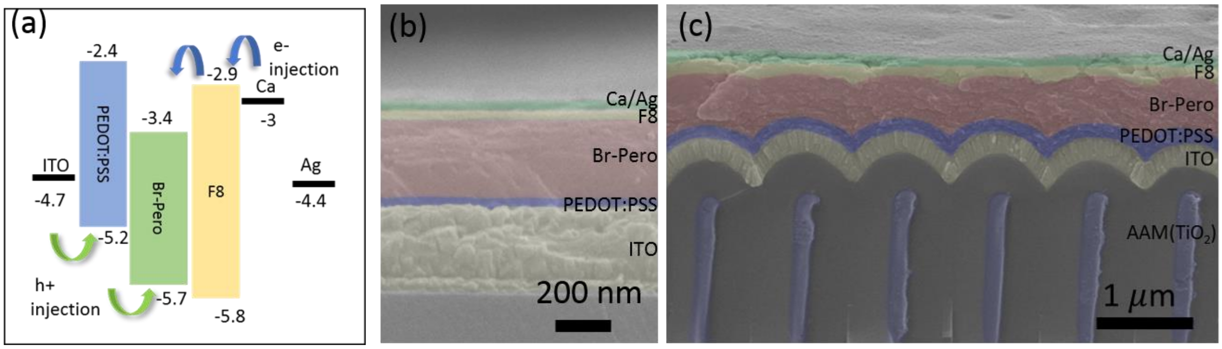

Supplementary Figure 2. (a) Band energy diagram of the device structure. Cross-sectional SEM images of (b) planar, and (c) P1000 AAM. For planar device, from bottom to top are: ITO, PEDOT: PSS, Br-Pero, F8 and Ca/Ag. For nanostructured devices, from bottom to top are: AAM with TiO_2, ITO, PEDOT: PSS, Br-Pero, F8 and Ca/Ag.

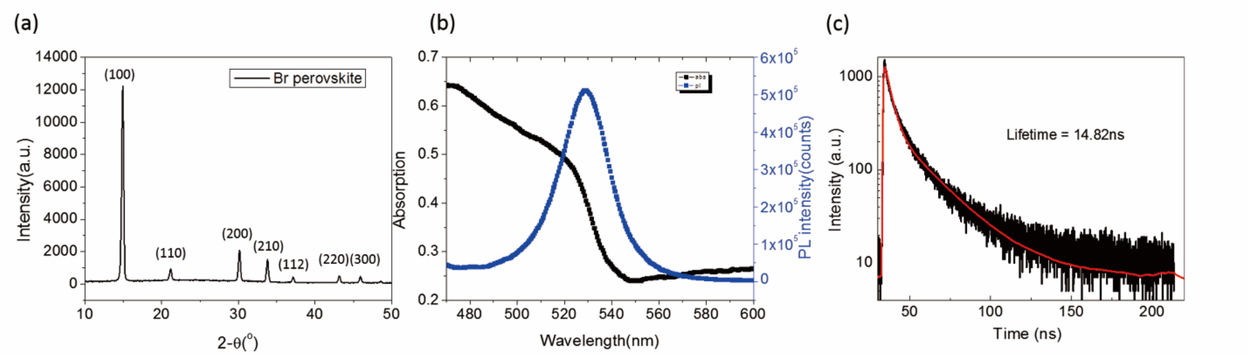

Supplementary Figure 3. (a) X-ray diffraction (XRD), (b) UV-visible and photoluminescence spectrums and (c) time-resolved PL (TRPL) of the $MAPbBr_3$ thin film.

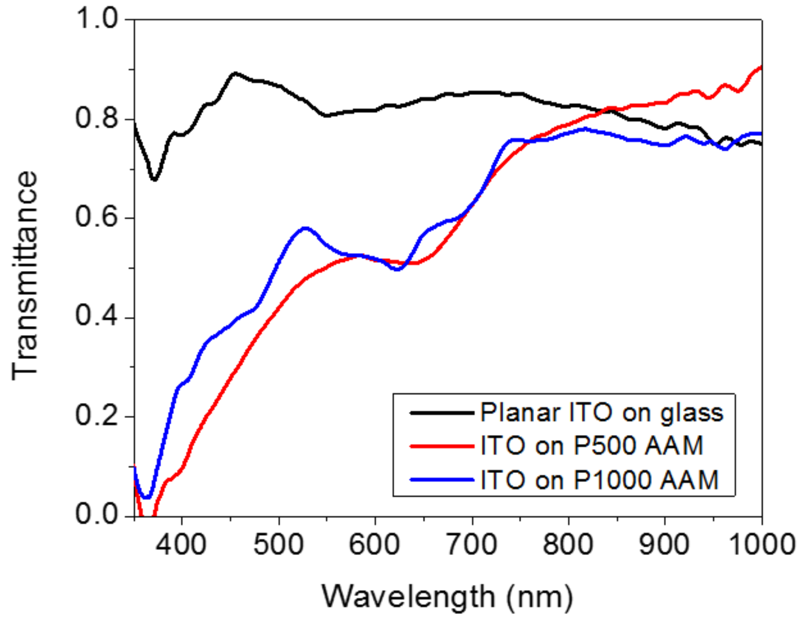

Supplementary Figure 4. Transmittance of the thin film (TF), P500 AAM and P1000 AAM substrates with ITO. In order to achieve a good sheet resistance, we used a relatively low O_2 flow rate (<1sccm) for sputtering ITO on P500 and P1000 nanostructured substrate thus compromised transmittance to certain degree. The sheet resistance is ~ 50 Ω/□ for ITO glass and ~ 90 Ω/□ for our sputtered ITO.

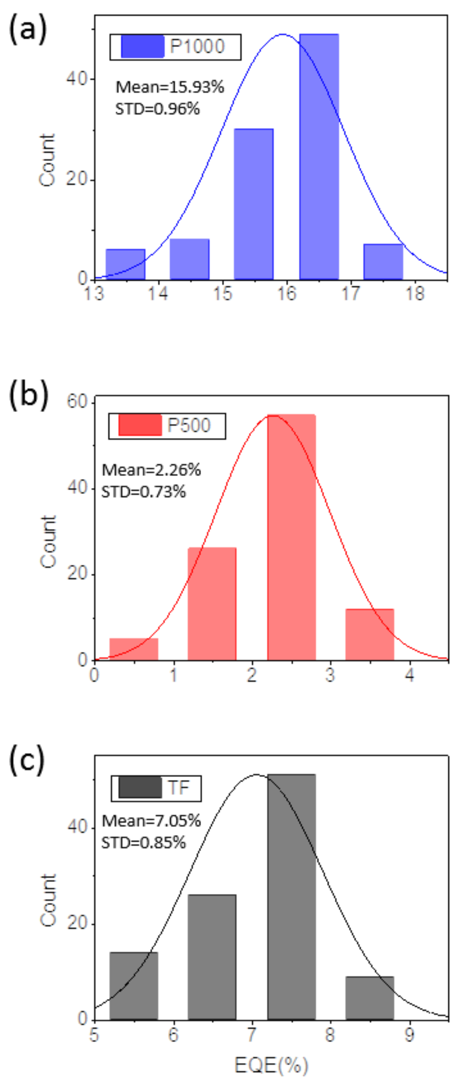

Supplementary Figure 5. Histogram of the EQE distribution for (a) P1000 AAM, (b) P500 AAM and (c) thin film (TF) devices. For each kind of device, ~100 devices were counted and the histogram of the EQE distribution is plotted. The P1000 AAM device has an average EQE of 15.93% with 0.96% standard deviation. The P500 AAM device has an average EQE of 2.26% with 0.73% standard deviation. And the thin film device has 7.05% average EQE with 0.85% standard deviation.

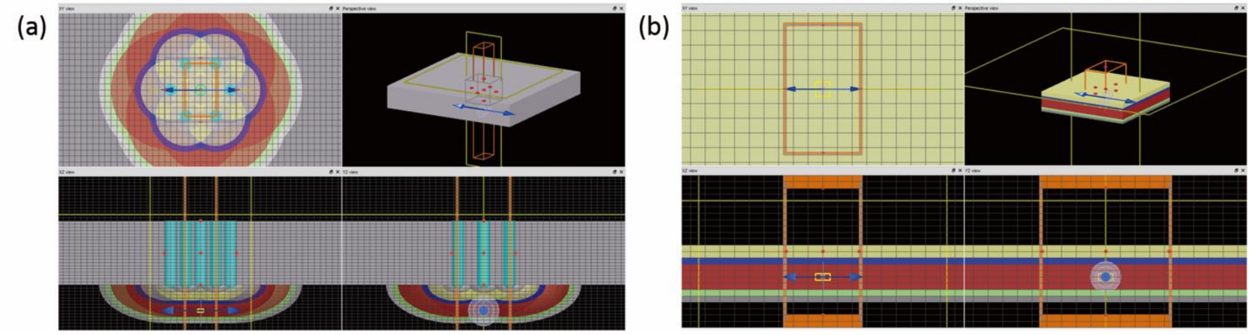

Supplementary Figure 6. Simulation model for (a) nanophotonic device and (b) planar device. Ag thickness is 100 nm. F8 thickness is 100 nm. Perovskite thickness is 400 nm. PEDOT: PSS thickness is 100 nm. AAM are hexagonal pores with pitch 500 nm, 1000 nm and 1500 nm. Pore sizes are 0.2×pitch, 0.4×pitch, 0.6×pitch and 0.8×pitch. AAM pores are filled with dielectric materials with index= 1, 1.5, 2.0 and 2.6. The simulation background is n=1.5, which is close to the epoxy refractive index and glass refractive index.

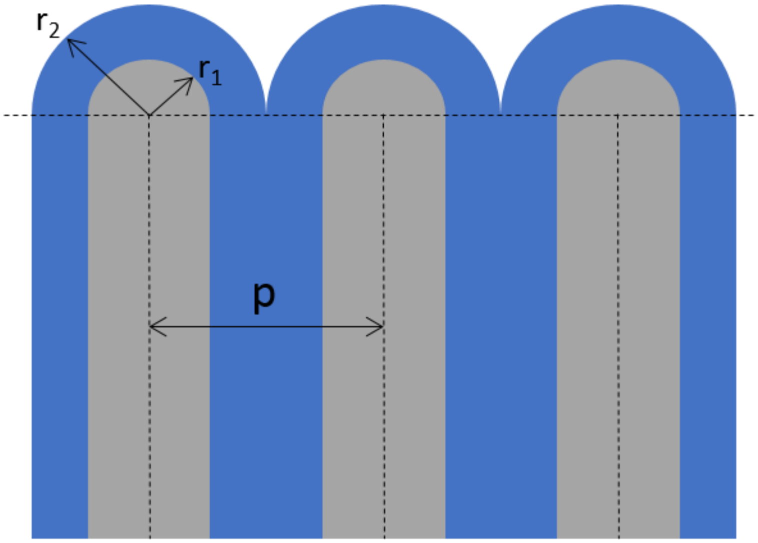

Supplementary Figure 7. Diagram of AAM cross-section. The DP ratio is defined as diameter of AAM pores divided by the AAM pitch. The AAM dome shape is determined by the AAM geometry. The pitch p is the x-axis in Fig. 3, and DP ratio is $\frac{2r_1}{p}$. The nanodome diameter r_2 is half of the pitch p.

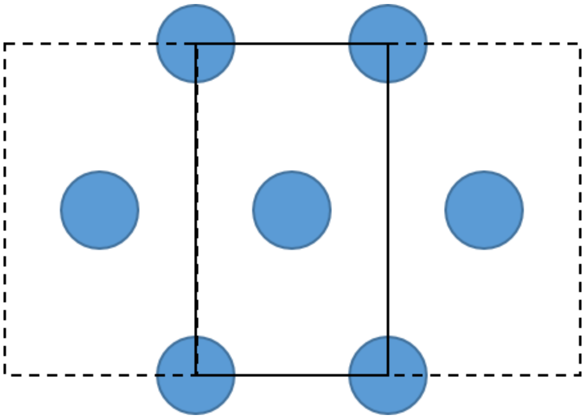

Supplementary Figure 8. Top view of the unit cell of the hexagonal AAM arrays.

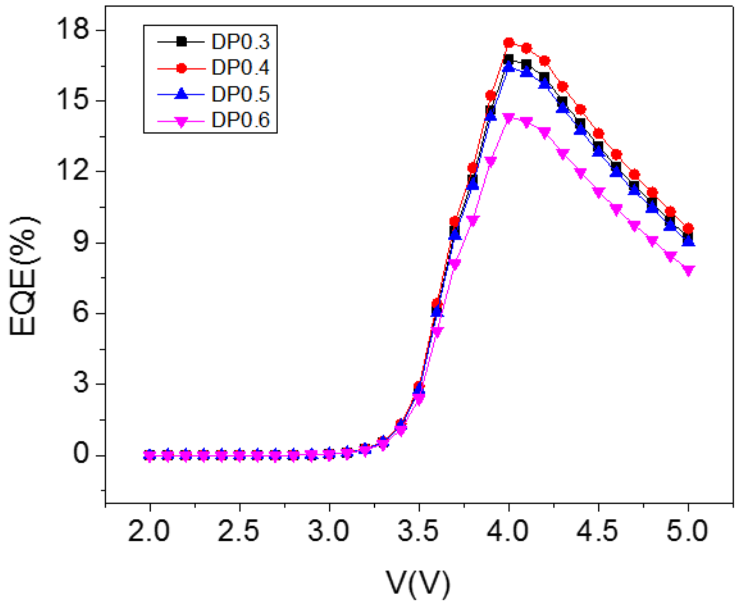

Supplementary Figure 9. EQE of the P1000 AAM devices with different DP ratios (diameter/pitch). Here the geometry effect of AAM substrate on LED device performance has been studied, and we changed not only the pitches, but also the diameters. Because P1000 AAM yielded the best EQE, the P1000 AAM was chosen for diameter effect study. Diameter-pitch (DP) ratio 0.3, 0.4, 0.5 and 0.6 corresponds to AAM pore size of 300 nm, 400 nm, 500 nm and 600 nm, respectively. The pore size can be easily controlled by the wet chemical etching time. And it was found the peak EQE for DP0.3, DP0.5 and DP0.6 are 0.96 times, 0.94 times and 0.82 times of the optimized DP0.4, which is quite consistent with our simulation result shown in Fig. 3a.

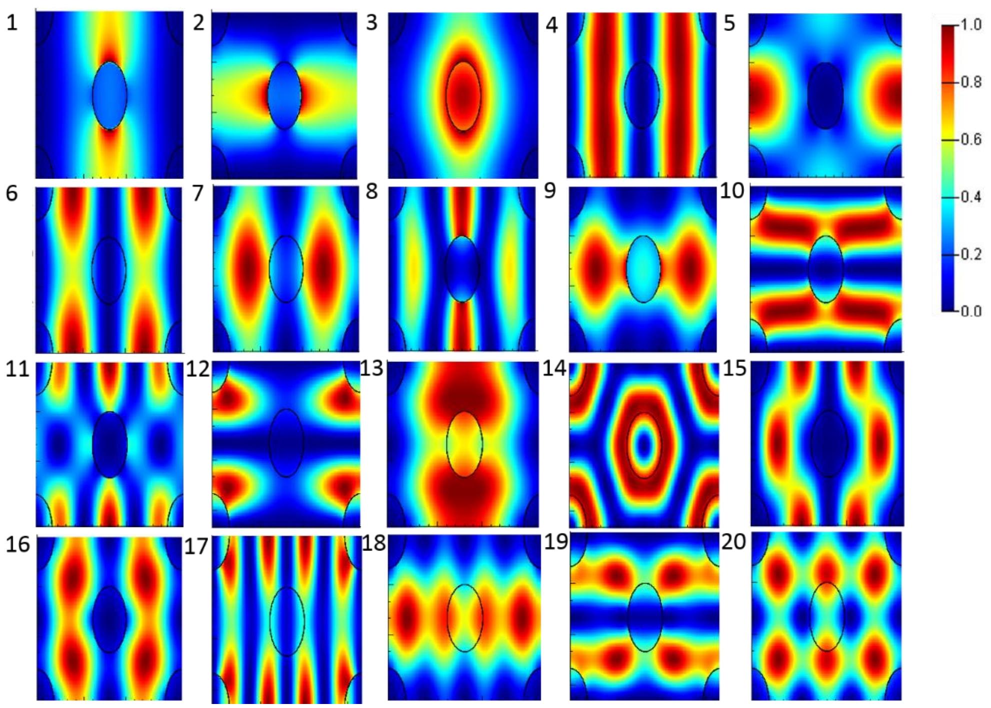

Mode list

mode #	effective index	wavelength (μm)	loss (dB/cm)	TE polarization fraction (Ey)	waveguide TE/TM fraction (%)
1	0.9887907	0.55	0.00000	1	99.93 / 27.9
2	0.0000000+2.021041i	0.55	2.0054e+006	98	99.29 / 35.08
3	0.0000000+2.142806i	0.55	2.1262e+006	26	43.76 / 99.99
4	0.0000000+2.615832i	0.55	2.5956e+006	0	99.97 / 40.43
5	0.0000000+2.615965i	0.55	2.5958e+006	75	99.97 / 40.43
6	0.0000000+3.800295i	0.55	3.7709e+006	1	63.82 / 46.1
7	0.0000000+3.949497i	0.55	3.9190e+006	37	44.43 / 65.58
8	0.0000000+4.390716i	0.55	4.3568e+006	2	94.03 / 46
9	0.0000000+4.926245i	0.55	4.8882e+006	73	48.3 / 99.59
10	0.0000000+5.181848i	0.55	5.1418e+006	99	99.54 / 47.05
11	0.0000000+5.182258i	0.55	5.1422e+006	25	99.54 / 47.05
12	0.0000000+5.421014i	0.55	5.3791e+006	99	90.13 / 47.34
13	0.0000000+5.644051i	0.55	5.6004e+006	11	45.62 / 84.28
14	0.0000000+6.049259i	0.55	6.0025e+006	50	100 / 47.59
15	0.0000000+6.104129i	0.55	6.0570e+006	1	59.35 / 48.37
16	0.0000000+6.227489i	0.55	6.1794e+006	20	47.46 / 60.46
17	0.0000000+6.668309i	0.55	6.6168e+006	6	73.35 / 48.26
18	0.0000000+6.823455i	0.55	6.7707e+006	79	47.19 / 72.19
19	0.0000000+7.040783i	0.55	6.9864e+006	94	59.68 / 48.6
20	0.0000000+7.199315i	0.55	7.1437e+006	52	47.73 / 58.66

Supplementary Figure 10. Modes in P100 AAM.

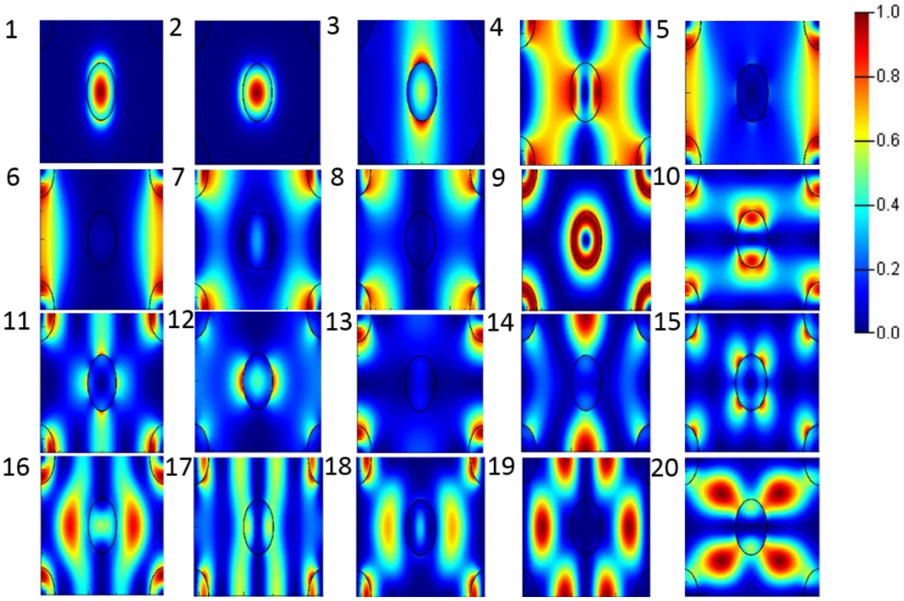

mode #	effective index	wavelength (μm)	loss (dB/cm)	TE polarization fraction (Ey)	waveguide TE/TM fraction (%)
1	2.110475	0.55	0.00000	1	84.81 / 90.97
2	2.106008	0.55	0.00000	99	83.71 / 90.39
3	1.745150	0.55	0.00000	29	91.96 / 99.7
4	1.725583	0.55	0.00000	6	99.51 / 80.62
5	1.725560	0.55	0.00000	72	99.51 / 80.62
6	1.712287	0.55	0.00000	85	98.07 / 78.95
7	1.688815	0.55	0.00000	8	96.23 / 78.55
8	1.632907	0.55	0.00000	14	87.11 / 87.26
9	1.585553	0.55	0.00000	50	100 / 54.03
10	1.563575	0.55	0.00000	89	97.48 / 63.39
11	1.563499	0.55	0.00000	29	97.48 / 63.4
12	1.560616	0.55	0.00000	70	68.38 / 97.55
13	1.558674	0.55	0.00000	71	90.53 / 68.62
14	1.483008	0.55	0.00000	21	88.35 / 77.69
15	1.420469	0.55	0.00000	48	74.43 / 74.11
16	1.358772	0.55	0.00000	68	81.75 / 66.28
17	1.324705	0.55	0.00000	19	72.85 / 76.07
18	1.295030	0.55	0.00000	25	80.93 / 61.19
19	1.243725	0.55	0.00000	1	71.81 / 78.01
20	1.166243	0.55	0.00000	92	58.88 / 82.17

Supplementary Figure 11. Modes in P500 AAM.

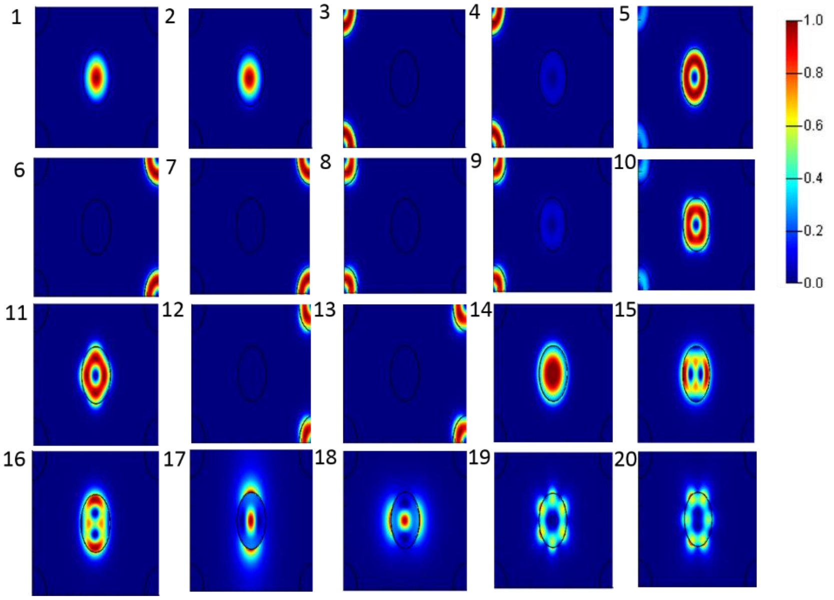

Mode list

mode #	effective index	wavelength (µm)	loss (dB/cm)	TE polarization fraction (Ey)	waveguide TE/TM fraction (%)
1	2.438285	0.55	0.00000	100	93.02 / 95.86
2	2.438285	0.55	0.00000	0	93.02 / 95.86
3	2.229115	0.55	0.00000	50	100 / 78.5
4	2.229110	0.55	0.00000	50	100 / 78.5
5	2.229097	0.55	0.00000	50	100 / 78.49
6	2.226451	0.55	0.00000	51	100 / 78.22
7	2.226445	0.55	0.00000	51	100 / 78.22
8	2.170782	0.55	0.00000	50	82.44 / 90.1
9	2.170773	0.55	0.00000	50	82.44 / 90.09
10	2.170753	0.55	0.00000	50	82.43 / 90.09
11	2.170651	0.55	0.00000	50	82.44 / 90.09
12	2.167306	0.55	0.00000	49	82.42 / 90.03
13	2.167295	0.55	0.00000	49	82.42 / 90.02
14	2.160293	0.55	0.00000	50	75.43 / 100
15	1.900818	0.55	0.00000	44	93.71 / 71.74
16	1.900618	0.55	0.00000	56	93.66 / 71.71
17	1.807054	0.55	0.00000	1	88.1 / 92.65
18	1.800462	0.55	0.00000	99	85.7 / 91.1
19	1.795529	0.55	0.00000	52	75.13 / 84.16
20	1.795151	0.55	0.00000	47	75.55 / 84.03

Supplementary Figure 12. Modes in P1000 AAM.

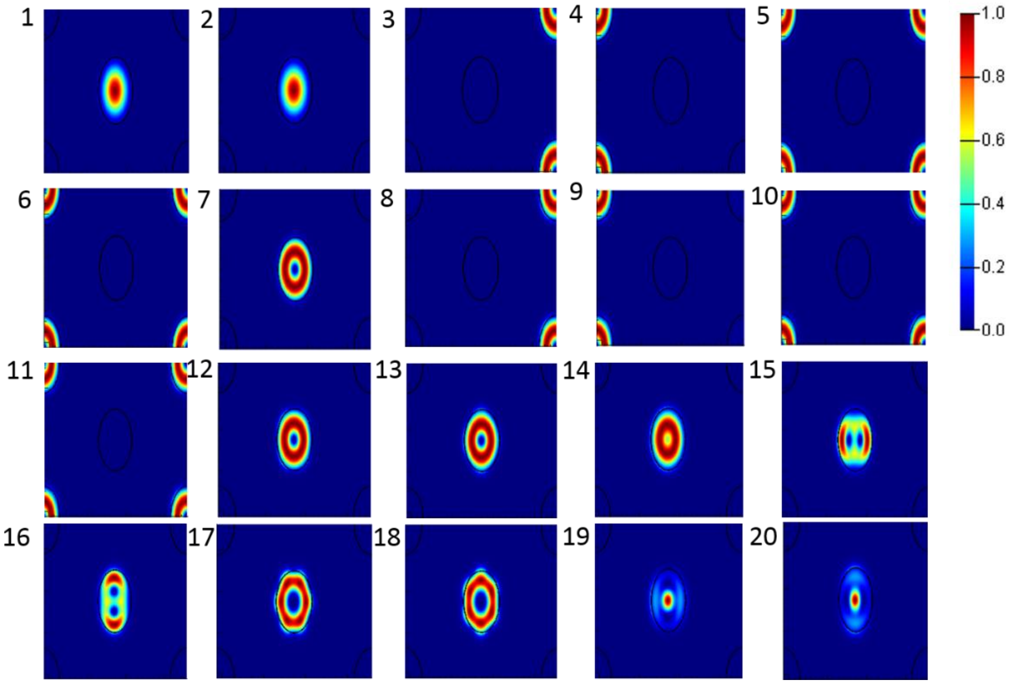

Mode list

mode #	effective index	wavelength (µm)	loss (dB/cm)	TE polarization fraction (Ey)	waveguide TE/TM fraction (%)
1	2.521873	0.55	0.00000	100	96.64 / 97.74
2	2.521873	0.55	0.00000	0	96.64 / 97.74
3	2.414815	0.55	0.00000	50	100 / 88.07
4	2.414815	0.55	0.00000	50	100 / 88.07
5	2.414815	0.55	0.00000	50	100 / 88.07
6	2.414815	0.55	0.00000	50	100 / 88.07
7	2.414813	0.55	0.00000	50	100 / 88.07
8	2.396359	0.55	0.00000	50	91.12 / 94.5
9	2.396359	0.55	0.00000	50	91.12 / 94.5
10	2.396359	0.55	0.00000	50	91.12 / 94.5
11	2.396359	0.55	0.00000	50	91.12 / 94.5
12	2.396357	0.55	0.00000	50	91.12 / 94.5
13	2.396318	0.55	0.00000	50	91.12 / 94.5
14	2.387252	0.55	0.00000	50	85.17 / 100
15	2.248417	0.55	0.00000	43	95.92 / 82.21
16	2.248416	0.55	0.00000	57	95.92 / 82.21
17	2.220904	0.55	0.00000	50	83.64 / 90.5
18	2.220904	0.55	0.00000	50	83.64 / 90.5
19	2.166446	0.55	0.00000	96	78 / 95.12
20	2.166446	0.55	0.00000	4	78 / 95.13

Supplementary Figure 13. Modes in P1500 AAM.

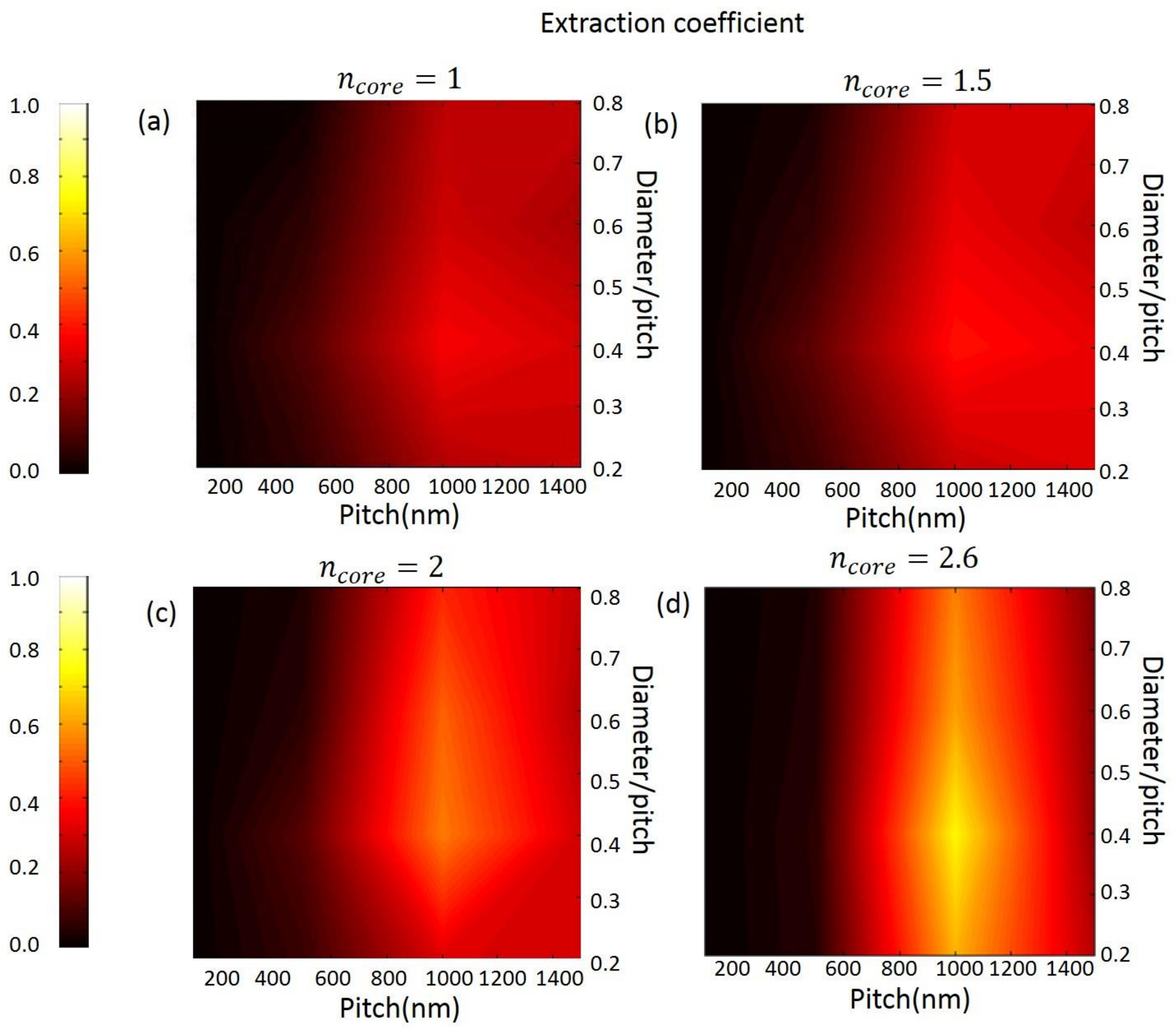

Supplementary Figure 14. Extraction coefficient for AAM core with different refractive indexes. $n_{\mathrm{core}} = 1$ corresponds to the case without TiO_2 and $n_{\mathrm{core}} = 2.6$ corresponds to the case with TiO_2. Theoretically, all materials with a refractive index 2.6 can replace the TiO_2. To verify the core refractive index effect, we added in two more simulations, namely, $n_{\mathrm{core}} = 1.5$ and $n_{\mathrm{core}} = 2.0$. We didn't simulate refractive higher than 2.6 because very few materials can have such high refractive index. Comparing Supplementary Figure 14(a)-(d), we found that $n_{\mathrm{core}} = 2.6$ performs the best. Intriguingly, the optimized structure is always the 1000 nm pitch with 0.4 aspect ratio. The best extraction coefficient is 0.359, 0.39, 0.55 and 0.736 for $n_{\mathrm{core}} = 1$, 1.5, 2 and 2.6, respectively.

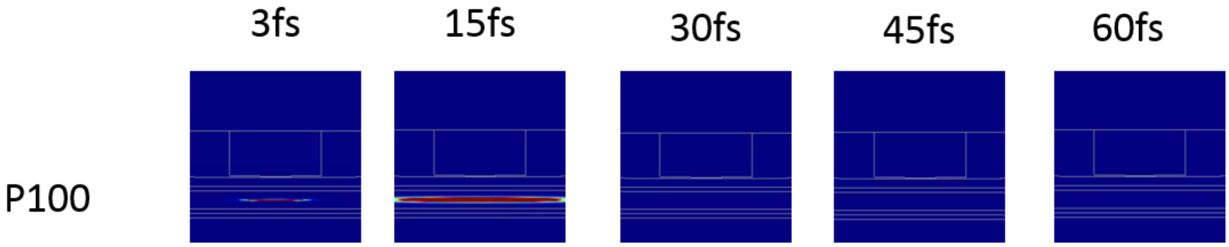

Supplementary Figure 15. Time domain *E* field evolution of P100 AAM. Light is propagating upwards.

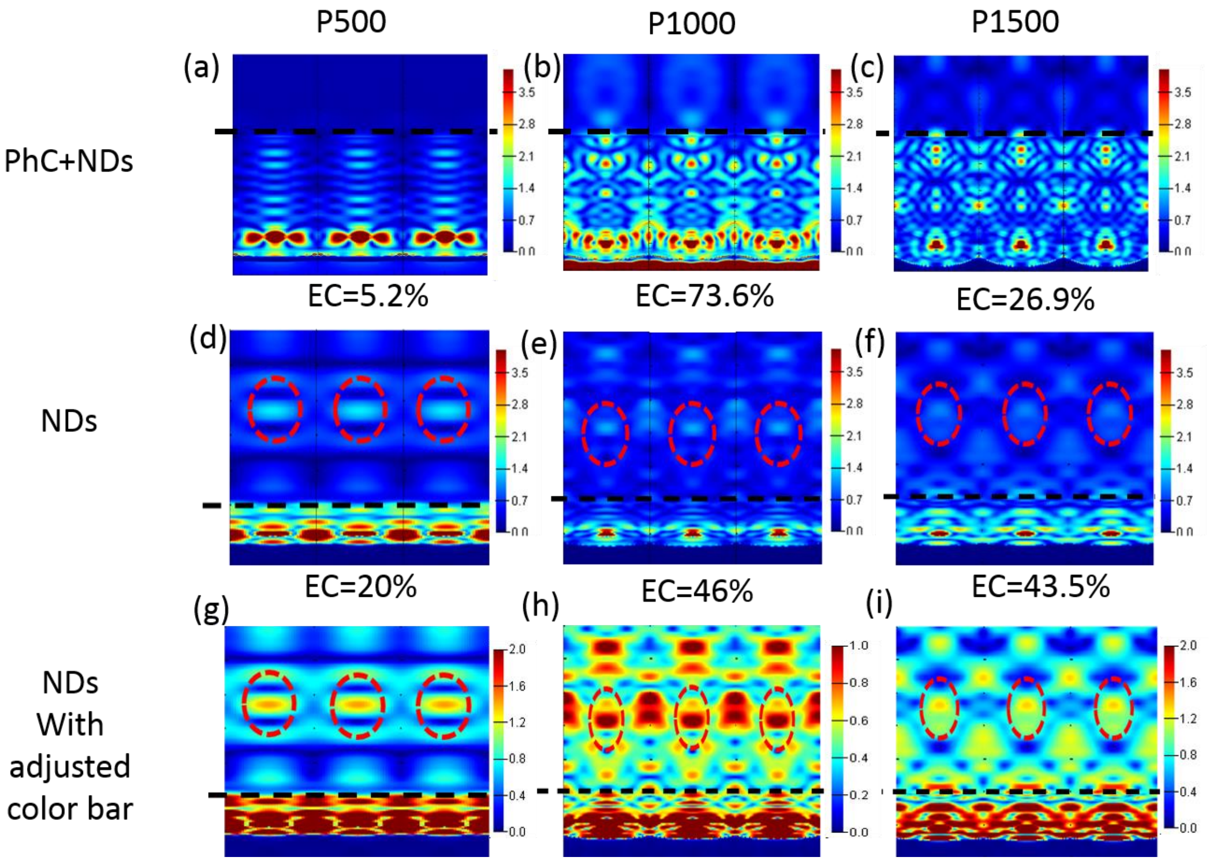

Supplementary Figure 16. E^2 intensities of (a)P500, (b) P1000 and (c) P1500 AAM devices with photonic crystal (PhC) and nanodomes (NDs). E^2 field intensities of (d)P500, (e) P1000 and (f) P1500 AAM devices with only NDs. E^2 field intensities with adjusted color bars to show the focus point of (g)P500, (h)P1000 and (i)P1500 AAM devices with only NDs. Extraction efficiency (EC) for different cases are also listed below each image. Dash lines are the interface between device and environment (UV epoxy, n=1.5).

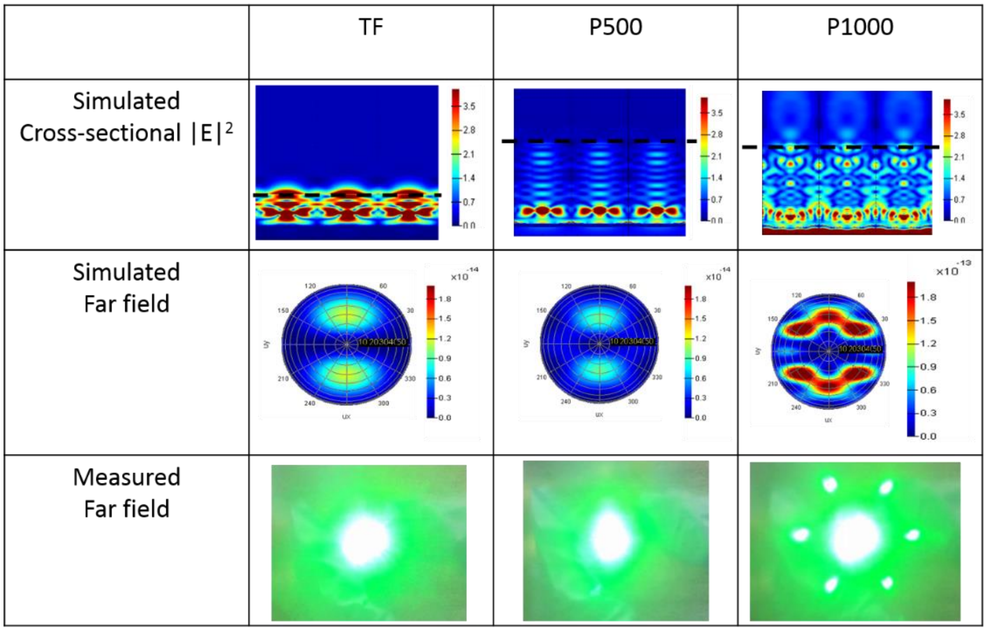

Supplementary Figure 17. Comparison of simulated cross-sectional E^2, simulated far field and measured far field of thin film (TF), P500 AAM and P1000 AAM devices.

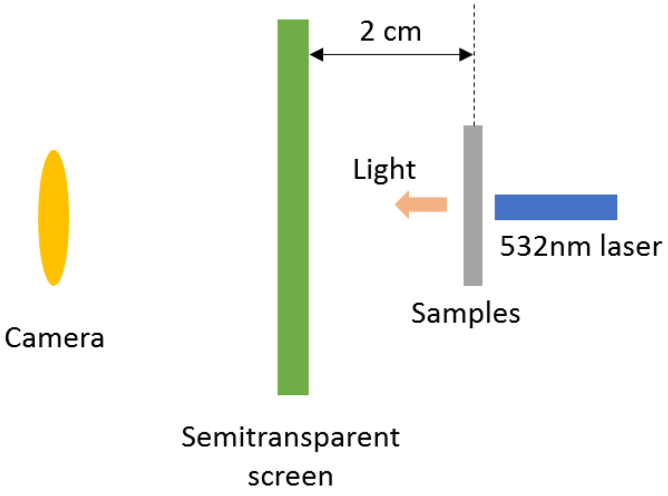

Supplementary Figure 18. Diagram of the far field measurement for thin film and nanophotonic devices.

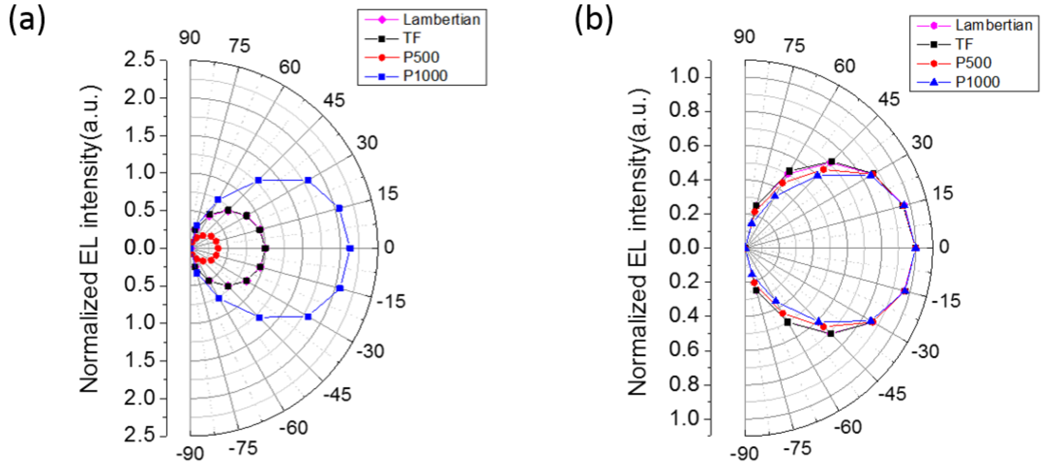

Supplementary Figure 19. Angular emission of the Lambertian profile, our thin film (TF) device, P500 AAM device and P1000 AAM device. (a) All intensities are normalized to the EL intensity of 0° emission of the TF device. (b) EL intensities are normalized to the EL intensity of 0° of each kind of device itself.

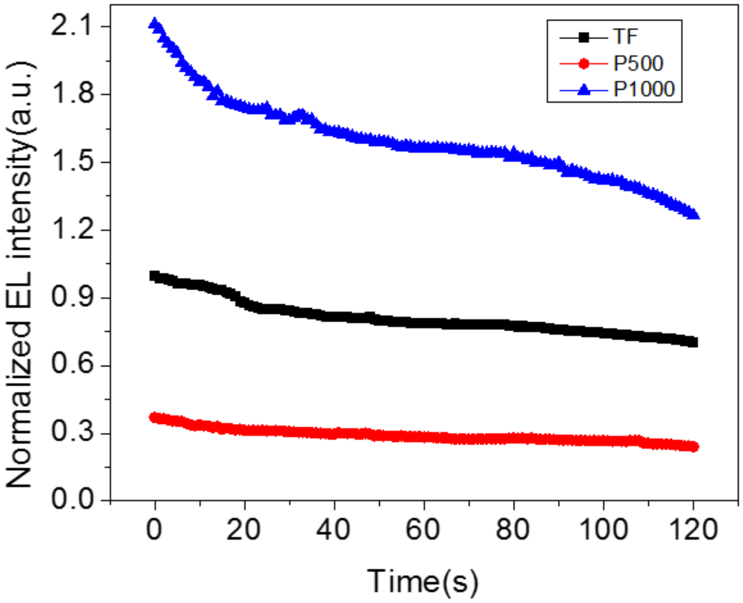

Supplementary Figure 20. Stability test of the thin film (TF), P500 AAM and P1000 AAM devices. The device stability was evaluated using EL intensity decay for two minutes with 4V driving voltage. Note that the driving current density 50 mA cm^{-2} and luminance of 10,000 cd m^{-2} is much higher than many reported results[1, 2]. It can be seen that the normalized EL for TF device dropped from 1 to 0.7, the normalized EL for P500 AAM dropped from 0.37 to 0.24, and the normalized EL for P1000 AAM dropped from 2.11 to 1.27. Currently, all of our devices are not packaged, and the measurements were performed in ambient condition with 75% humidity, this also caused device performance decay.

Supplementary Tables

Supplementary Table 1. Summary of the maximum EQE of the high-performance green light perovskite LED and their emitting materials. For those estimated light extraction efficiencies, we simply calculated by dividing maximum EQE by PLQY.

**Articles**	**Max. EQE**	**Year**	**Emitting materials**	**PLQY**	**Light extraction efficiency**
K. Lin *et. al.*[1]	20.3%	2018	$CsPbBr_3$ /MABr quasi-core/shell	80%	25.4% (estimated)
Z. Xiao *et. al.*[3]	9.3%	2016	BABr:$MAPbBr_3$	Not reported	Not mentioned
H. Cho *et. al.*[4]	8.53%	2015	$MAPbBr_3$	Not reported	Not mentioned
X. Yang *et. al.*[5]	14.36%	2018	$PEA_2(FAPbBr_3)_{n-1}PbBr_4$	73.8%	19.6% (estimated)
M. Ban *et. al.*[6]	15.5%	2018	$PEA_2Cs_{n-1}Pb_nBr_{3n+1}$	70%	22% (estimated)

Supplementary Notes

Supplementary Note 1

X-ray diffraction (XRD) was carried out to characterize the composition and crystallinity of the films, and the results are shown in Supplementary Figure 3. The peaks at 15°, 21°, 30° and 34° matched well with the (100), (110), (200) and (210) planes of cubic Br-Pero crystals, confirming the presence of cubic Br-Pero crystals. The carrier lifetime of Br-Pero was characterized to be 14.82 ns by time resolved photoluminescence (TRPL) with results shown in Supplementary Figure 3c. The lifetime here is long among reported Br-Pero and it indicates a good perovskite material quality.

Supplementary Note 2

From the simulation results, it was found that 1000 nm pitch and 0.4 aspect ratio is the optimized geometry. Here we calculated the photonic crystal filling fraction and effective refractive index for this geometry. The diagram for the hexagonal AAM is shown in Supplementary Figure 8, the solid line rectangular is one unit-cell. Then the filling fraction of the high-index core is given as,

$$F = \frac{2\times(\pi {r_{\mathrm{channel}}}^2)}{pitch\times\sqrt{3}pitch}. \quad (1)$$

Given that $\frac{r_{\mathrm{channel}}}{pitch} = 0.4$, F is then calculated to be 0.145.

Therefore, the effective refractive index of the nanophotonic substrate is given as,

$$n_{\mathrm{eff}} = \sqrt{Fn_{\mathrm{TiO2}}^2 + (1-F)n_{\mathrm{AAO}}^2}. \quad (2)$$

Given that $n_{\mathrm{TiO_2}} = 2.6$ and $n_{\mathrm{AAO}} = 1.7$, n_{eff} can be calculated to be 1.86.

Therefore, the optimized structure is a positive photonic crystal with fill fraction of 0.145 and effective index of 1.86.

Supplementary Note 3

Supplementary Figure 10-13 show the modes in different geometry AAMs. Note that here we only show the guided modes in the TiO_2 core. And only the first 20 modes (from fundamental modes to high order modes) are calculated. As for P100 AAM (Supplementary Figure 10), there is no fundamental TE or TM mode. And the only guided mode 3 has an imaginary effective index and suffers from very high loss. This phenomenon supports our argument that the P100 AAM supports no guided modes, and therefore the light extraction for P100 AAM is very poor. As for the P500 AAM (Supplementary Figure 11), there are 3 guided modes (mode 1, 2 and 9) in the center TiO_2

core. Mode 1 is TM_0 fundamental mode, mode 2 is TE_0 fundamental mode, and mode 9 is hybrid mode.

As for the P1000 AAM (Supplementary Figure 12), there are 8 guided modes (mode 1, 2, 5, 10, 11, 14, 15, 16). Mode 1 is TE_0 fundamental mode, and mode 2 is TM_0 fundamental mode. Other modes are hybrid modes. Mode 17, 18, 19 and 20 can be classified as leaky modes. These leaky modes are favorable for light to be extracted out and become radiation propagation. Mode 17 is TM_1 mode, and mode 18 is TE_1 mode. As for the P1500 AAM (Supplementary Figure 13), there are 12 guided modes (mode 1, 2, 7, 12, 13, 14, 15, 16, 17, 18, 19 and 20). Mode 1 is TE_0 fundamental mode, and mode 2 is TM_0 fundamental mode. Mode 19 is TE_1 mode, and mode 20 is TM_1 mode. Other modes are hybrid modes.

The mode analysis for P1000 and P1500 AAM also supports our point in the main text that the P1500 AAM allows more guided modes than P1000 AAM. As only the guided modes scattered by/ interacted with the nanostructures can be extracted out, therefore a proper amount of guided modes is required. This also supports the result that P1500 AAM is not as good as P1000 AAM in terms of light extraction.

Supplementary Note 4

The nanodomes in our structures are working as focusing lens to couple light into the photonic crystal optical antennas. The focusing effect can be supported by the Supplementary Figure 16 g-i clearly. Note that the extraction efficiencies for P500, P1000 and P1500 AAM devices with only nanodomes (NDs) are 20%, 46% and 43.5%, respectively. Intriguingly, P500 and P1500 AAM devices show better ECs with only NDs than with both photonic crystal (PhC) and NDs. This is another strong support that the P1000 PhC has better capability of extracting the light out than P500 and P1500 PhCs. With the optimal P1000 AAM PhC, the EC has been increased from 46% with only NDs to 73.6% with both PhC and NDs. As a result, the combination of NDs with PhCs show its better strength in light extraction than NDs only, but only when the geometry of the PhC is properly designed.

Supplementary Note 5

In order to understand the function of the photonic crystal part without nanodomes, we also studied the light propagation through the photonic crystals. In this study, we input plane wave into the photonic crystals with different pitches and calculate the near field light output and the light extinction (1-transmittance). Fig. 6 show the near field of the P500, P1000 and P1500 photonic

crystals, respectively. For P500 and P1500, it can be seen that the light energy is confined inside the TiO_2 channels. The field pattern of P1000 photonic crystal near field (Fig. 6b) shows a scattering resonance (leaky mode). [7] The scattering resonance is also an evidence of the optical antennas effect which can effectively convert the confined light energy to propagating radiation, and the antennas effect can also be supported by the extinction spectrum pass band centering at 530 nm (EL peak).[8, 9] And this pass band has a quality factor of 30 ($f_1 = 5.77 \times 10^{14} Hz, f_2 = 5.58 \times 10^{14} Hz, f_c = 5.675 \times 10^{14} Hz, Q = \frac{f_c}{f_1 - f_2}$). As for the P500 photonic crystal, the extinction (Fig. 6d) shows a typical interference pattern, which indicates the standing wave (guided modes) is confined inside TiO_2 channels, which can also be supported by Supplementary Figure 16a and Fig. 6a. And if we look at the extinction of P1500 (Fig. 6f), 530 nm is at the edge of one pass band, which means this wavelength is not in the working range of P1500 optical antennas, and that is why most of the light energy is confined in the TiO_2 channels, as shown in Fig. 6c. Basically, this explains why adding photonic crystals can help the light extraction of P1000 AAM device but not for P500 and P1500 AAM device.

Supplementary Note 6

In order to perform far field measurement, a semitransparent screen was placed 2 cm far from our samples and a 532 nm laser was used as light source in order to excite strong enough far field. We performed the far field measurement and added the result as Supplementary Figure 17. In order to approximate the linear polarized light source in the simulation and also in order to excite strong enough far field, a laser was chosen to excite far field for thin film and nanophotonic samples. Especially, the measured far field pattern of the P1000 AAM matches very well with the simulation results. On the other hand, the TF and P500 samples do not have clear far field pattern in both simulation and measurements. Intriguingly, when we rotate the laser, the far field pattern of our P1000 sample also rotates. Considering our LED device generates random and non-polarized light when electrically turned on, it’s difficult to see the far field pattern from the electrically pumped device. Similar situation has also been discussed in Z. Khokhar *et. al.*’s work, far field EL of their photonic crystal LED has no clear diffraction spots because light was uniformly generated all over the LED surface and couples collectively to the quasi-photonic crystal structures[10].

Supplementary Note 7

It can be seen that the angular emission spectra, no matter thin film or nanostructured ones, basically follow the trend of the Lambertian profile (Supplementary Figure 19b). Generally, the overall EL intensity of P1000 AAM device is two times of that of the TF one. Note that the nanostructured devices show a marginally reduced EL intensity at emission angle larger than 30°, which is more obvious in Supplementary Figure 19a. This can be understood because the large angle emission is focused by the AAM dome shapes which can be interpreted as focusing lens, as can be supported by our light propagation videos in supplementary. The AAM domes help couple the light from perovskite layer into the AAM channels (TiO_2) and form guided modes. Basically, these guided modes propagate vertically. Thanks to the scattering effect, the guided modes can be extracted out by a proper geometry (P1000), therefore, the vertically propagating light will become random directions again after being scattered by the nanophotonic substrates.

Supplementary Note 8

For our 1st generation device, we used pure $MAPbBr_3$ without any material engineering process. Both device efficiency and PLQY were not high, but the enhancement factor is 5.06, which is close to the 7 times enhancement from simulation result of EC. For the 2nd generation device, we optimized the perovskite layer by crystal pining method[4]. The device performance was enhanced by the PLQY for TF and nanostructured devices started to show some difference. We attribute this to the surface roughness of nanostructured devices which is not favorable for film flatness during spin coating. Afterward, device efficiency and our thin film device baseline was enhanced to 8.19% which is close to the 9.3% record of this particular material system.[3] The performance improvement was due to the additive of long-chain group material BABr which helps to further reduce crystal size and improve the perovskite flatness/smoothness[3]. Then the difference of PLQY between thin film and nanostructured devices became more obvious. All these results indicate that the conventional material engineering methods targeting at crystal pining and perovskite flatness will show less effectiveness when applied to a nanostructured substrate. However, we still see an improvement in the device EQE by using the nanophotonic substrate (P1000) even though the PLQY was reduced to less than half of that of the thin film device. This can be a strong evidence of the important role of nanophotonic substrate.

Moreover, the ITO transmittance on AAMs is not as good as our commercial ITO glass. As shown in Supplementary Figure 4, the ITO glass shows 80% transmittance at 550 nm while the ITO on

AAM substrate shows only 50% transmittance. This is because the non-ideality of our sputtering process. In order to achieve good electrical conductance, O_2 flow rate was relatively low (less than 1sccm) during sputtering which compromised transmittance to certain degree. This ITO transmittance loss also made the EQE enhancement of our real device not as high as the modeled results.

Supplementary References

1. Lin K, *et al.* Perovskite light-emitting diodes with external quantum efficiency exceeding 20 per cent. *Nature* **562**, 245-248 (2018).

2. Wang N, *et al.* Perovskite light-emitting diodes based on solution-processed self-organized multiple quantum wells. *Nat. Photonics* **10**, 699 (2016).

3. Xiao Z, *et al.* Efficient perovskite light-emitting diodes featuring nanometre-sized crystallites. *Nat. Photonics* **11**, 108 (2017).

4. Cho H, *et al.* Overcoming the electroluminescence efficiency limitations of perovskite light-emitting diodes. *Science* **350**, 1222-1225 (2015).

5. Yang X, *et al.* Efficient green light-emitting diodes based on quasi-two-dimensional composition and phase engineered perovskite with surface passivation. *Nat. Commun.* **9**, 570 (2018).

6. Ban M, *et al.* Solution-processed perovskite light emitting diodes with efficiency exceeding 15% through additive-controlled nanostructure tailoring. *Nat. Commun.* **9**, 3892 (2018).

7. Cao L, Fan P, Brongersma ML. Optical coupling of deep-subwavelength semiconductor nanowires. *Nano lett.* **11**, 1463-1468 (2011).

8. Schuller JA, Taubner T, Brongersma ML. Optical antenna thermal emitters. *Nat. Photonics* **3**, 658 (2009).

9. Bharadwaj P, Deutsch B, Novotny L. Optical antennas. *Adv. in Opt. & Photonics* **1**, 438-483 (2009).

10. Khokhar AZ, *et al.* Emission characteristics of photonic crystal light-emitting diodes. *Appl. opt.* **50**, 3233-3239 (2011).
