# Supplementary material for: Efficient metal halide perovskite light-emitting diodes with significantly improved light extraction on nanophotonic substrates
Source: Nat Commun. 2019 Feb 13;10:727. doi: 10.1038/s41467-019-08561-y (PMC6374404; doi:10.1038/s41467-019-08561-y)
Supplement: Supplementary file 3 — Description of Additional Supplementary Files [file 41467_2019_8561_MOESM3_ESM.pdf]

Description of Additional Supplementary Files

Supplementary Movie 1
Light propagation for planar device.

Supplementary Movie 2
Light propagation for P100 AAM device.

Supplementary Movie 3
Light propagation for P500 AAM device.

Supplementary Movie 4
Light propagation for P1000 AAM device.

Supplementary Movie 5
Light propagation for P1500 AAM device.
